# Role of vasopressin in the treatment of anaphylactic shock in a child undergoing surgery for congenital heart disease: a case report

**DOI:** 10.1186/1752-1947-2-36

**Published:** 2008-02-05

**Authors:** Luca Di Chiara, Giulia V Stazi, Zaccaria Ricci, Angelo Polito, Stefano Morelli, Chiara Giorni, Ondina La Salvia, Vincenzo Vitale, Eugenio Rossi, Sergio Picardo

**Affiliations:** 1 Department of Pediatric Cardiology and Cardiac Surgery, Bambino Gesù Hospital, Rome, Italy

## Abstract

**Introduction:**

The incidence of anaphylactic reactions during anesthesia is between 1:5000 and 1:25000 and it is one of the few causes of mortality directly related to general anesthesia. The most important requirements in the treatment of this clinical condition are early diagnosis and maintenance of vital organ perfusion. Epinephrine administration is generally considered as the first line treatment of anaphylactic reactions. However, recently, new pharmacological approaches have been described in the treatment of different forms of vasoplegic shock.

**Case presentation:**

We describe the case of a child who was undergoing surgery for ventricular septal defect, with an anaphylactic reaction to heparin that was refractory to epinephrine infusion and was effectively treated by low dose vasopressin infusion.

**Conclusion:**

In case of anaphylactic shock, continuous infusion of low-dose vasopressin might be considered after inadequate response to epinephrine, fluid resuscitation and corticosteroid administration.

## Introduction

The incidence of anaphylactic reactions during anesthesia is between 1:5000 and 1:25000 and it is one of the few causes of mortality directly related to general anesthesia [1]. The most important requirements in the treatment of this clinical condition are early diagnosis and maintenance of vital organ perfusion. Epinephrine administration is generally considered as the first line treatment of anaphylactic reactions [1]. However, recently, new pharmacological approaches have been described in the treatment of different forms of vasoplegic shock [2]. We describe a case in which low dose vasopressin promply re-established hemodynamic stability in a vasoplegic state due to an anaphylactic reaction that was refractory to epinephrine infusion.

## Case presentation

A 6-year-old 18 kg male with a ventricular septal defect and history of asthma was scheduled for surgical correction. The patient had never undergone general anesthesia and had a past medical history of bronchial asthma treated with inhaled salbutamol. General anesthesia was induced with 0.2 mg/kg of midazolam, 0.2 mg/kg cisatracurium besylate and 0.5 mcg/kg remifentanil. Intravenous general anesthesia was maintained with continuous infusion of remifentanil (0.25–0.5 mcg/kg/min), cisatracurium besylate (0.2 mg/kg/hr) and midazolam (0.2 mg/kg/hr). Continuous monitoring included electrocardiogram, invasive systemic arterial pressure (SAP) and central venous pressure (CVP), transcutaneous arterial oxygen saturation (SatO2), end tidal CO_2 _(Et CO_2_), cerebral saturation detected by near infrared spectroscopy monitoring (cSvO2), and peripheral, rectal and nasopharyngeal temperature. After induction vital signs were stable: SAP 80/40 mmHg, heart rate (HR) 110 beats/min, SatO_2 _98%, CVP 8 mmHg, EtCO_2 _34 mmHg, cSvO2 80%.

Antibiotic therapy (amoxicillin/clavulanate potassium) and methylprednisolone (30 mg/kg) were administered as routine before sternotomy incision. Before starting cardiopulmonary bypass (CPB), 380 UI/kg of heparin were given and after about 60 seconds a sudden cutaneous rush and hemodynamic instability with severe hypotension appeared: SAP decreased to 40/25 mmHg, HR raised to 180 bpm, CVP fell to 1 mmHg, cSvO2 fell below 40%. Airway pressure increased to 5.06 kPa with the clinical finding of bilateral pulmonary wheezing. In order to re-establish hemodynamic stability, volume resuscitation was started (30 ml/Kg) and two intravenous (iv) boluses of 500 mcg of epinephrine (by institutional protocol: 25 mcg/kg every 5 minutes) were given while oxygen inspiratory fraction was increased to 1. CPB was instituted in 5 minutes in order to improve patient organ perfusion: CPB pump flow initially set to 150 ml/kg/min (corresponding to a cardiac index of 3.3 L/min/m^2^) generating a perfusion pressure of 20 mmHg with systemic vascular resistances index (SVRI) of 470 dyne*s/cm^5^/m^2^. Anaphylactic reaction to heparin with a distributive shock was strongly suspected. The finding of metabolic acidosis (pH 7.23) with increased lactate levels (9 mmol/L) suggested poor tissue perfusion due to severe hypotension-low perfusion pressure with inadequate oxygen delivery to peripheral tissues. Initial management of shock consisted of moderately hypothermic (30°C) high-flow CPB (220 ml/kg/min) with hematocrit increased from 30% to 35% by transfusion of 200 ml of packed red blood cell. Moreover, epinephrine infusion was started at a dose to 0.1 mcg/kg/min in order to achieve a perfusion pressure of 40 mmHg.

Metabolic acidosis progressively improved (pH = 7.38) with an initial reduction in plasma lactate levels (5.1 mmol/L). When vital parameters seemed adequately stable, the surgical procedure was performed with a CPB time of 25 minutes. During this time, the epinephrine infusion could not be stopped and the first weaning from CPB failed because of severe hypotension (mean SAP = 30 mmHg) despite epinephrine administration being titrated up to 0.3 mcg/kg/min. Arginine-vasopressin (Pitressin; Monarch Pharmaceuticals, Bristol, United Kingdom) infusion was started at a rate of 0.0003 U.I./Kg/min. Within 5 minutes, a pump flow at 100 ml/kg/min generated a perfusion pressure of 40 mmHg with a significant rise of SVRI to 1400 dyne*s/cm^5^/m^2^.

Epinephrine infusion was immediately reduced to 0.05 mcg/kg/min and the patient was successfully weaned from CPB with stable hemodynamic parameters. Protamine was administered without any adverse effect. After admission to the pediatric cardiac intensive care (PCICU), the patient's hemodynamics were stable and urine output was 3 ml/kg/h without any electrolytic disorder. Lactate levels returned to normal values within 6 hours. Vasopressin was progressively reduced by 0.0001 U.I./Kg/min every 2 hours, controlling SAP to more than 80/40 mmHg, and stopped after 6 hours infusion. Epinephrine was reduced and stopped in 12 hours with the same hemodynamic goal. The patient was extubated 12 hours after the surgical procedure and discharged from PCICU after 24 hours. No adverse effects due to the vasopressin administration were reported.

## Discussion

Anaphylactic and anaphylactoid reactions during anesthesia are generally caused by neuromuscular blocking agents, some general anesthetics, antibiotics, blood products, opioids, latex and rarely by anticoagulant agents such as heparins [3]. Cardiovascular collapse due to anaphylaxis is a vasodilatory shock, characterized by an abrupt fall in systemic vascular resistance, enhanced vascular permeability, intravascular volume depletion and metabolic acidosis with hyperlactatemia.

Metabolic acidosis is mainly derived from poor tissue perfusion due to severe hypotension and low perfusion pressure rather than inadequate systemic oxygen delivery only. The distribution of cardiac output to the various organs and to the regulation of the microcirculation that can be substantially altered in several conditions (i.e. distributive shock) where local control of vascular tone is altered and the formation of edema may contribute to damage to the distribution of blood flow. Multiple mediators from mast cells, such as kinins, leukotrienes and prostanoids, are implicated in promoting vasodilatation, but histamine seems to play the major role [4]. Stimulation of histamine-H1 receptors on endothelium cells activates both the nitric oxide (NO) and the prostacycline mediated vasodilating pathways [5]. Activation of inducible NO synthase (iNOS) is a major contributor to both vasodilatation and resistance to the catecholamine vasopressor effect. NO decreases myosin light chain phosphorylation and activates calcium-sensitive (K_Ca_) and adenosine triphosphate-sensitive (K_ATP_) potassium channels in the plasma membrane of vascular smooth-muscle cells through both direct and cyclic guanosine monophosphate (cGMP) pathways [4]. Potassium channel activation results in K efflux, cellular hyperpolarization, closure of the voltage-gate calcium channels and blunting of the intracytosolic calcium rise sustaining vasoconstriction. Finally, prolonged low systemic hypoperfusion with tissue hypoxia and lactic acidosis can maintain all the described pathophysiologic mechanisms and induce a relative deficiency in vasopressin plasma concentration further amplifying the vasoplegic scenario [5]. Despite the presence of histamine receptors the heart is not the target organ and cardiac abnormalities during anaphylactic reaction are due to severe impairment in perfusion pressure or to side effects of administered catecholamines [6]. Epinephrine has been widely accepted to be the standard medical therapy to reverse cardiovascular collapse in anaphylaxis. Because of its α and β adrenergic effects, epinephrine inhibits further vasodilating mediator release from basophils and mast cells, reduces bronchonstriction, increases vascular tone and improves cardiac output. Nevertheless, in the complex pathophysiologic mechanism of anaphylactic shock, inotropic resistance has been described and epinephrine may fail to reverse vasodilation [7,8] while sustaining undesidered effects related to increased myocardial oxygen consumption. Recently, the successful use of vasopressin to treat septic and postcardiotomy shock has been documented [2,9] and pathophysiologic considerations supporting its role in the treatment of vasodilatory shock have been demonstrated. Vasopressin inhibits the synthesis of iNOS, blunts the increase in cGMP induced by NO and directly inactivates K_ATP _channels in vascular smooth muscle [10]. Moreover, vasopressin is able to enhance endogenous catecholamine-induced vasoconstriction [11]. Despite the evidence that anaphylaxis causes a clinical picture of intense vasodilation, there are few cases reporting vasopressin administration to treat anaphylactic shock [12]. To our knowledge this is the first case report documenting the evidence of efficacy of vasopressin administration in anaphylactic shock in pediatric cardiac surgery. Our patient did not respond adequately to volume expansion and epinephrine infusions. Our decision to start CPB might have been questionable since the patient might have been stabilized with epinephrine and vasopressin and the case rescheduled.

Nevertheless, our choice was made in order to urgently restore adequate ventilatory parameters and to improve organ perfusion within the extracorporeal circuit before the clinical picture of severe vasoplegic shock was completely defined. It must be considered that CPB might also have initially worsened the clinical picture since, once the inflammatory system is activated, it is likely that CPB will add further activation. However, only the administration of low dose vasopressin was effective in restoring adequate systemic vascular resistance and allowed for a successful CPB weaning and stable postoperative hemodynamic parameters. Given the the existing controversy on which agent should be preferably used in case of vasoplegic shock [13], our decision to use vasopressin was related to other recent available experiences [12], the above described pharmacological rationale and the choice of avoiding escalating therapy with alpha agonists. This pharmacological approach allowed us to titrate the drug to the minimum required dose and avoided side effects reported with high vasopressin doses such as reduction of diuretic output and hyponatremia [14]. The adequacy of tissue peripheral perfusion was confirmed by the postoperative normalization of plasma lactate levels.

## Conclusion

In case of anaphylactic shock, continuous infusion of low-dose vasopressin might be considered in the treatment algorithm after inadequate response to epinephrine, fluid resuscitation and corticosteroid administration. Vasopressin may help to promptly and effectively restore hemodynamic stability and adequate systemic oxygen delivery before the disastrous effects of massive distributive shock can lead to severe organ hypoperfusion and cell death.

## Abbreviations

**SAP**: invasive systemic arterial pressure; **CVP**: central venous pressure; **SatO2**: trascutaneous arterial oxygen saturation; **Et CO**_2_: end tidal CO_2_; **cSvO2**: cerebral saturation (detected by near infrared spectroscopy monitoring); **HR**: heart rate; **CPB**: cardiopulmonary bypass; **SVRI**: systemic vascular resistances index; **NO**: nitric oxide; **iNOS**: inducible Nitric Oxide synthase; **K**_**Ca**_: calcium-sensitive potassium channels; **K**_**ATP**_: adenosine triphosphate-sensitive potassium channels; **cGMP**: cyclic guanosine monophosphate.

## Competing interests

The author(s) declare that they have no competing interests.

## Authors' contributions

LDC, ZR and GVS have made substantial contributions to the conception and design, acquisition of data, and analysis of data. AP, SM, CG, OLS, VV and ER have been involved in drafting the manuscript or revising it, and for critical review of important intellectual content. SP gave final approval of the version to be published. All authors read and approved the final manuscript

## Consent

Written informed consent was obtained from the patient's relatives for publication of this case report. A copy of the written consent is available for review by the Editor-in-Chief of this journal.
